# Integrated Evaluation of Electrical Breakdown Strength and Mechanical Properties of 3D-Printed Polymers, Supplemented by ImageJ-Based Surface Damage Analysis

**DOI:** 10.3390/polym18111345

**Published:** 2026-05-29

**Authors:** Anıl Şahin

**Affiliations:** Aircraft Technology, Vocational College of Technical Sciences, Trakya University, Edirne 22100, Türkiye; anilsahin@trakya.edu.tr

**Keywords:** 3D printing polymers, mechanical strength, breakdown strength, electrical insulation, high voltage, image analysis

## Abstract

Fused deposition modelling (FDM) is being increasingly considered for manufacturing electrically insulating components with complex geometries; however, the relationship between mechanical performance and dielectric breakdown behaviour of common printable polymers remains insufficiently understood. This study investigates the electrical insulation potential of five FDM-printed thermoplastic polymers—ABS, PLA, PETG, ASA, and PC/ABS—by evaluating their dielectric breakdown strength and mechanical properties. Specimens were fabricated using fused deposition modelling and tested according to standardised procedures: dielectric breakdown strength was measured in accordance with IEC 60243, and tensile properties were determined following ASTM D638. Surface damage produced during breakdown events, including holes and carbonised regions, was quantified using ImageJ analysis. The results were evaluated comparatively to identify the advantages and limitations of each material for electrical insulation applications. Among the tested materials, PLA exhibited the highest mechanical strength and the lowest surface damage area, whereas ABS demonstrated the highest dielectric breakdown strength. These findings highlight the trade-offs between mechanical and dielectric performance in material selection for 3D-printed insulators, and they demonstrate that ImageJ-based damage analysis serves as a valuable supplementary tool for characterising breakdown behaviour.

## 1. Introduction

Additive manufacturing (AM) refers to a technique for building an object layer by layer using computer-aided design (CAD) data [1]. Additive manufacturing offers exceptional design flexibility, particularly for the production of parts with complex geometries [2,3] and customised products [1]. Moreover, by significantly reducing material waste and shortening production times, it thus contributing to sustainability goals [3,4,5]. One of the most widely used 3D printing methods is fused deposition modelling (FDM). In FDM, a thermoplastic filament is melted and extruded through a nozzle to build a 3D object layer by layer on a build platform [6,7,8].

The 3D printing of electrical and electronic components represents a significant innovation in the field of engineering [2,6,7,9]. This technology enables the production of various components such as connectors, antennas, and sensors that are difficult or impossible to manufacture using conventional methods, while offering advantages such as material diversity, complex geometry capability, and reduced production times [6,9]. A number of researchers have explored the development of printed electronics using three primary classes of materials: conductors, semiconductors, and insulators [9]. The authors build on the many advantageous aspects of 3D printing technology and demonstrate through their work that it is possible to print a wide variety of electronic components. Tan et al. [10], for example, recently provided an overview of the state-of-the-art additive manufacturing in electronic systems. Tan et al. showed through their review that four different methods (fused deposition modelling or FDM, Ink Jet Printing, Aerosol Jet Printing, and Electrohydrodynamic Jet Printing) had been successfully used to create the individual building blocks of electronic systems including conductive traces, resistors, capacitors, inductors, antennas, and sensors [10]. Using multi-material approach to 3D printing allows the creation of even more complicated systems, such as field effect transistors, quantum dot light emitting diodes, and three-dimensional interconnects [11]. Due to the wide variety of materials available with FDM, which is also known for being very inexpensive, this has become one of the most popular choices for creating electronic components using additive manufacturing [12].

The electrical breakdown process is when an insulator (dielectric) breaks down and loses its ability to prevent electricity from flowing through it once it exceeds a certain amount of voltage [13,14]. Electrical breakdown is extremely important for reliable device performance in the electrical and electronic industries [15,16]. Defects at the microscopic scale within a solid dielectric that can cause breakdown include surface defects, internal voids or holes and contamination. All these types of defects can initiate breakdown events. For printed parts made with FDM, defects such as rough surfaces, porous layers, and interfaces between printing layers can reduce electrical breakdown resistance [17]. Since most defects caused during the FDM printing process occur due to the layering process, they can be especially detrimental in high-voltage applications. Even minor defects can result in early insulation failures [18].

The mechanical properties of 3D-printed parts (tensile strength, compressive strength, flexural strength, hardness) are critical depending upon application requirements [19,20]. The orientation of the part on the build plate significantly influences mechanical performance because layers oriented parallel to or perpendicular to applied load induce anisotropy [21,22]. In addition to layer orientation, infill density has a large impact upon the mechanical properties of 3D-printed parts; typically, higher infill densities produce stronger and harder components [20,21]. A 100% infill is selected to maximise resistance to mechanical stresses [20].

Among additive manufacturing technologies, FDM is one of the most commonly employed processes to create polymer-based electrically insulated components because of its relatively low cost, availability, and wide variety of applicable materials. While this method produces products at a very low cost and has a great number of available materials, it is still necessary to consider several disadvantages. The FDM technology uses a layer-by-layer approach to fabricate parts. This creates non-uniform mechanical behaviour along different axes (anisotropy), uneven surfaces, and voids or air gaps between layers that will negatively impact the structural integrity and dielectric uniformity of fabricated parts. Initial studies focused on widely used FDM materials like acrylonitrile-butadiene-styrene (ABS) and poly(lactic acid) (PLA). Initial results indicated that the dielectric breakdown strength of both of these two materials exceeded 30 kV/mm when subjected to alternating currents (AC). It was observed that ABS provided better electrical and thermal stability than PLA. Additionally, PLA provided a dielectric constant of about 3.25. The dielectric constant may be decreased, and the mechanical properties of a part when the infill ratio may also be decreased, resulting in an increased void content. As a result, the polarisation capability of the material was diminished [17,23]. It has been reported that the voids produced during processing are responsible for significant portions of variation and reduced reliability in dielectric performance, limiting it in medium- and high-voltage applications [17]. Studies subsequent to the above referenced works investigated other candidates, such as polyethylene terephthalate glycol (PET-G), acrylonitrile styrene acrylate (ASA), and polypropylene (PP), concluding that the optimal material choice is dependent upon the applied voltage stress environment. For example, although PP exhibited excellent dielectric performance under unipolar dc and impulse voltages, the poor printability characteristics of PP on commercially available FDM printers greatly limited the practical application of PP. Conversely, ABS and PET-G displayed good performance under bipolar AC conditions. ASA was found to provide good balance of performance over all combinations of tested voltage stresses [24]. On top of this work, additional research was conducted validating the engineering feasibility of using FDM-printed thermoplastics for medium-voltage power electronic insulation. Specifically, researchers created functional insulation applications including 20 kV DC inductor bobbin assemblies. Simulations and experimental data verified the ability to use ABS under real-world operating conditions. Also, it was determined that increasing switching speeds can increase electrical stresses and emphasise the importance of evaluating both dielectric and mechanical performance in insulation design [18]. More recent research has evaluated bio-based and composite polymeric systems for high-voltage insulation applications. Unfortunately, prior to now, there has not been adequate breakdown strength or process-related void development to make them practical alternatives in demanding electrical insulation environments [25,26]. While substantial progress has been made with respect to examining dielectric breakdown behaviour and mechanical performance individually for FDM-printed thermoplastic insulators, there currently does not exist a comparative framework to integrate both criteria in standardised testing conditions for FDM-printed thermoplastic insulators. Furthermore, the dielectric and mechanical performance of FDM-printed polycarbonate/acrylonitrile butadiene styrene (PC/ABS) remains insufficiently explored, despite its promising potential for power electronics applications. In addition, surface damage resulting from dielectric breakdown events has yet to be systematically established as a quantitative diagnostic parameter for material selection in electrical insulation applications.

In this study, the mechanical properties and dielectric breakdown behaviour of FDM-printed solid insulating materials—namely ABS, PLA, PETG, ASA, and PC/ABS—were systematically investigated and compared under standardised testing conditions. Dielectric breakdown strength was measured in accordance with IEC 60243-1/2/3 [27,28,29], while tensile tests were conducted following ASTM D638-14 [30]. Surface damage induced by breakdown events was quantitatively characterised using ImageJ (1.52a) image analysis software. Based on the results, correlations between mechanical properties, breakdown strength, and surface damage characteristics were evaluated. To the best of the author’s knowledge, the dielectric breakdown behaviour and mechanical properties of FDM-printed thermoplastic insulators have not been jointly evaluated within an integrated comparative framework. This study addresses this gap by providing a comprehensive assessment of ABS, PLA, PETG, ASA, and PC/ABS under standardised conditions, and by proposing an approach for selecting the most suitable material with optimum properties for medium-voltage power electronic applications based on both dielectric and mechanical performance criteria.

## 2. Materials and Methods

This section outlines the properties of the polymer filaments used in the study and the printing parameters applied to each filament during 3D printing. Furthermore, it provides details of the methods employed in dielectric breakdown and tensile strength tests and information on the test setup used in the high-voltage laboratory.

### 2.1. Properties of Material

Filaments made from various materials and having different properties are commercially available for use in 3D printers. In this study, ABS, PLA, PETG, ASA, and PC/ABS filaments were selected because they exhibit higher electrical insulation properties than other available filaments. All filaments used in the experiments were supplied by Porima Polimer Teknolojileri A.Ş. (Yalova, Türkiye). All filaments have a diameter of 1.75 mm. The physical, thermal, and electrical properties of the filaments are presented in Table 1.

### 2.2. 3D Printing Process

**Figure 1:**
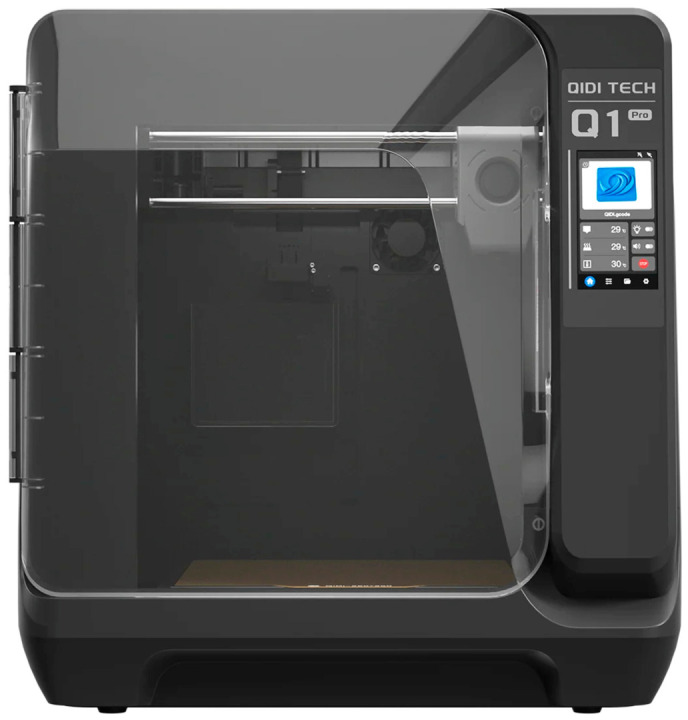
Enclosed Qidi Tech Q1 Pro CoreXY 3D printer.

A Qidi Q1 Pro model CoreXY FDM 3D printer, manufactured by DI JIA Technology Limited (Hong Kong, China), was used to produce all specimens for experimental studies. The printer’s enclosed architecture and active chamber heating capability provided controlled processing conditions during specimen production. Figure 1 shows the 3D printer used in this study, while detailed technical specifications are available in Ref. [32].

**Figure 2:**
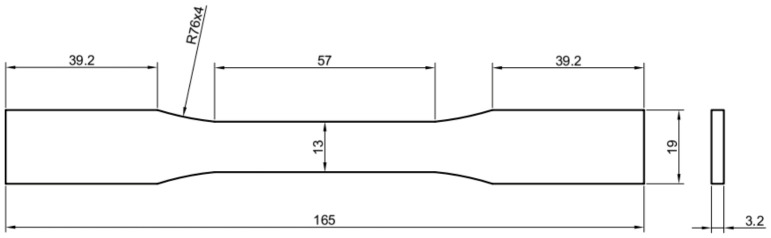
ASTM D638-14 Type I specimen dimensions, with all dimensions expressed in millimetres.

Two types of specimens were produced for use in dielectric breakdown strength tests and tensile tests. The specimens used in the dielectric breakdown strength tests were circular, with a diameter of 50 mm and a thickness of 1.2 mm, and the dog-bone specimens used in the tensile test were produced in accordance with the ASTM D638-14 (Type I) standard. The dimensions of the dog-bone specimens are given in Figure 2.

The specimens used in the experiments were modelled in 3D using CAD (FreeCAD 1.0.2) software and saved in STL file format. The STL files for the specimens were opened in the QidiStudio 1.2.0 slicing software, the printer type was selected, and the G-codes required for printing the specimens were generated using the printing parameters given in Table 2. The specimens were printed on the 3D printer using the obtained G-codes. Before starting all printing processes, the build plate calibration was performed automatically by the printer. During specimen printing, the environment housing the 3D printer was maintained at 23 °C.

**Figure 3:**
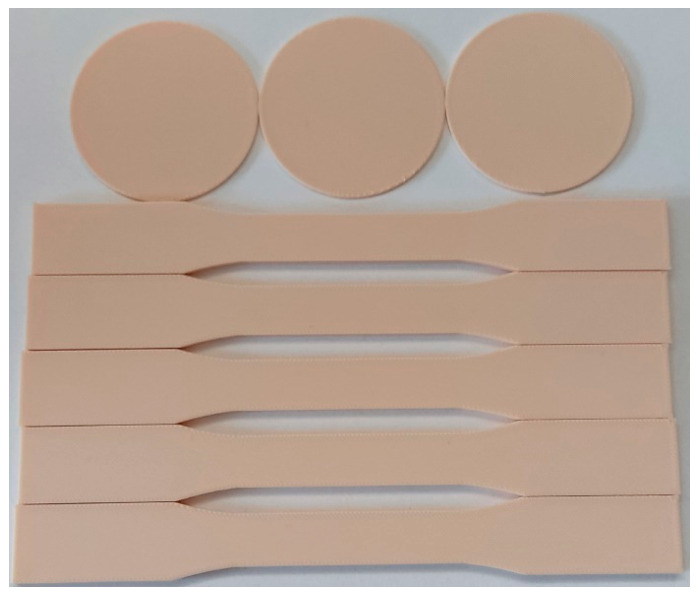
PLA tensile and electrical breakdown specimens.

ABS, PLA, PETG, ASA, and PC/ABS filaments were used to print the specimens. The filaments were dried before use to prevent printing issues and to standardise the printing process. The drying process was carried out in a Creality Space Pi filament-drying unit (Creality 3D Technology Co., Ltd., Shenzhen, China) at the temperatures and drying times specified in Table 3. The specimens produced by the printing process are shown in Figure 3.

### 2.3. Tensile Testing

**Figure 4:**
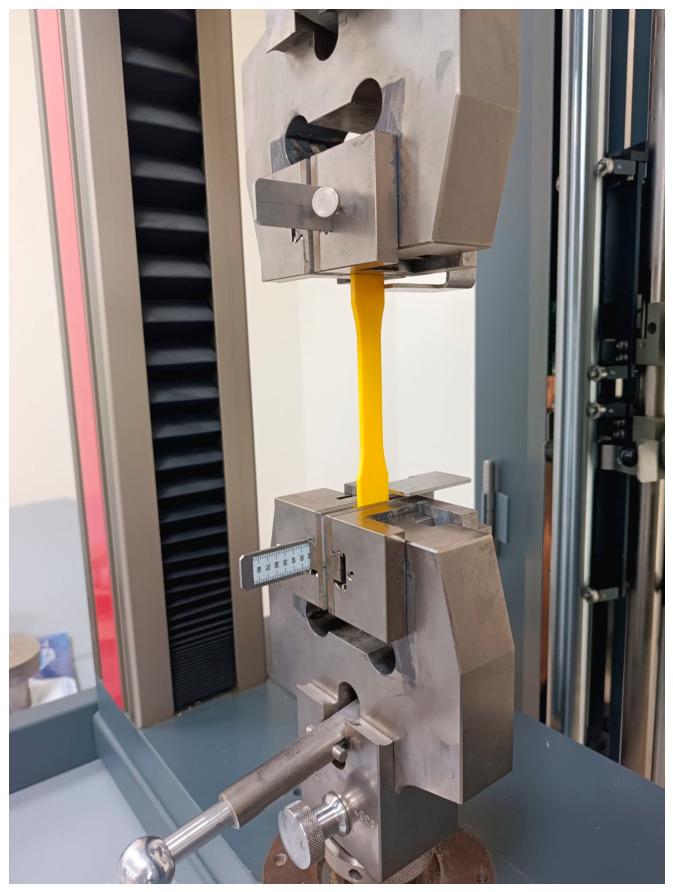
Tensile test.

Tensile tests were carried out in accordance with the ASTM D638-14 standard on a Zwick/Roell universal testing machine (Z020, ZwickRoell GmbH & Co. KG, Ulm, Germany) (Figure 4). All tests were conducted at an ambient temperature of 23 °C. The tensile tests were carried out at a crosshead speed of 5.0 mm/min using a 10 kN load cell. Five specimens were used per group, and the means and standard deviations were calculated.

### 2.4. Dielectric Breakdown Testing

**Figure 5:**
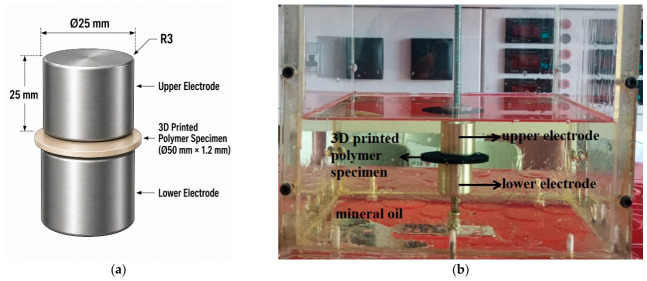
Experimental container: (**a**) electrode configuration; (**b**) electrode arrangement.

Various test methodologies, which are widely accepted in the literature and in industrial applications, are used to determine the dielectric strength and electrical breakdown characteristics of solid insulating materials. In this context, the IEC-60243-1/2/3 and IEC-60060-1/2 [33,34] standards provide specialised test procedures tailored to different voltage types and material properties, thereby achieving universal validity for the measurements. If the breakdown strength of the material under test is low and the electrical stress does not exceed the insulating capacity of the ambient air, conducting the tests under atmospheric conditions poses no technical problem. However, when high voltage levels are reached, particularly when there is a risk of strong surface discharges forming on the specimen, it is essential to modify the test environment to maintain measurement accuracy. In such conditions, insulating fluids with significantly higher dielectric strength—such as mineral oil, silicone fluid, or ester-based fluids—are preferred to prevent the discharge from jumping off the material surface and to directly identify the breakdown point within the material’s internal structure [35,36,37]. In the method detailed in Section 10 of the IEC 60243-1 standard, which is defined as the ‘Short-duration (rapid rise) test’, the test voltage applied to the specimen is increased from zero at a specified rate. Although the standard defines rise rates ranging from 100 V/s to 5000 V/s, the 500 V/s rise rate has become the most commonly used in laboratories worldwide, as it yields the most consistent and comparable results across different materials. The used in the experiments consists of a polymethyl methacrylate (Plexiglas) test container measuring 200 × 200 × 200 mm, selected for its insulating properties and ease of observation. The test container is shown in Figure 5. In the electrode configuration, which forms the heart of the system, a ‘cylinder-cylinder’ arrangement fully compatible with the relevant standards has been employed. This arrangement comprises two identical cylindrical electrodes, each 25 mm in diameter and 25 mm in height, with corners rounded to a radius of 3 mm to minimise localised field concentrations caused by edge effects; it is designed to measure the material’s dielectric strength with the highest precision.

**Figure 6:**
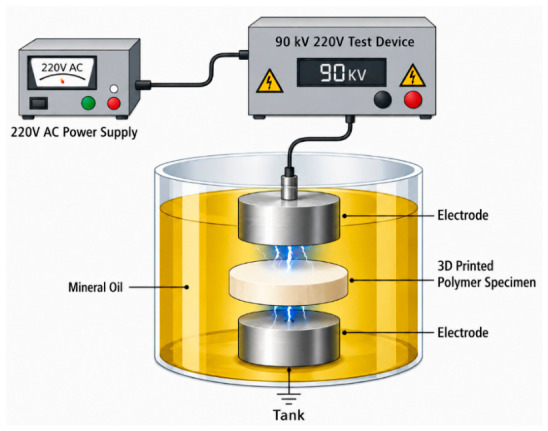
Connection diagram for breakdown strength tests.

In the tests conducted to determine the dielectric strength of insulation materials, a 90 kV AC voltage source was used, and the voltage rise rate was set to 500 V/s in accordance with standards. To prevent unwanted surface discharges during testing and maintain measurement accuracy, the electrode system and test specimens were immersed in a test chamber filled with Nytro Lyra X mineral oil. The electrical connection diagram and component layout of the test setup are presented in detail in Figure 6. Each experiment was repeated three times for each material.

## 3. Results and Discussion

### 3.1. Mechanical Properties of PLA, ABS, PETG, ASA, and PC/ABS Specimens

To quantitatively compare the mechanical performance of the investigated 3D-printed polymer specimens, tensile tests were conducted in accordance with ASTM D638-14. The resulting elasticity, strength, and deformation parameters are presented in Table 4.

**Figure 7:**
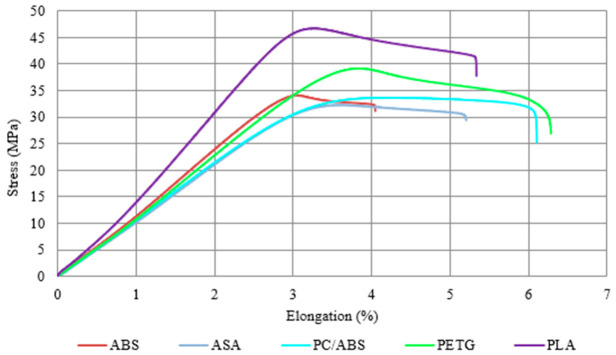
Representative stress–strain curves obtained from tensile tests for each filament type (*n* = 5). The endpoints of the curves indicate where the specimens fractured.

The representative stress–strain curves presented in Figure 7 and the mechanical properties listed in Table 4 show that the filaments under investigation exhibit distinctly different deformation behaviours, ranging from ductile to brittle. Differences in parameters, such as elastic modulus, maximum tensile stress, and elongation at break, indicate that the materials’ deformation and energy dissipation capacities vary significantly.

**Figure 8:**
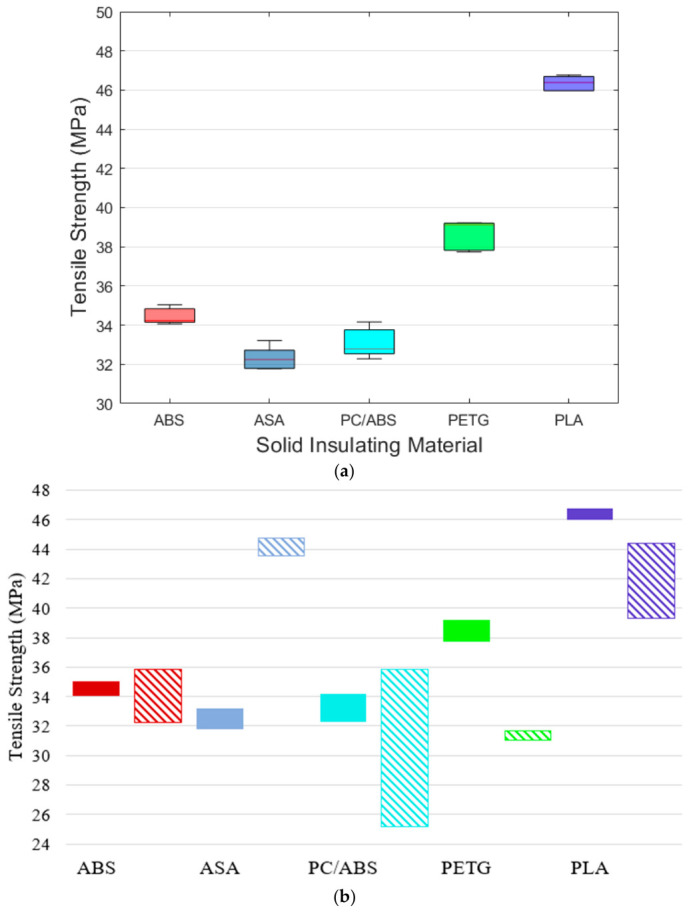
(**a**) Experimental tensile strength distribution of tested materials (*n* = 5); (**b**) comparison of experimentally obtained tensile strength values with the reported literature ranges for comparable FDM-printed thermoplastic materials ABS [38], ASA [39], PC/ABS [40], PETG [41] and PLA [42].

The mechanical properties shown in Table 4 provide statistical support for the deformation behaviour observed in Figure 7. PLA filament exhibited the highest tensile strength among the materials studied at 46.44 ± 0.36 MPa, while ASA had the lowest tensile strength at 32.32 ± 0.60 MPa. ABS (34.46 ± 0.42 MPa) and PC/ABS (33.10 ± 0.77 MPa) specimens exhibited similar tensile strength values. PETG, with a tensile strength of 38.63 ± 0.76 MPa, offered an intermediate level of mechanical strength within the group (Figure 8a). The tensile strength values obtained through experimentation generally fell within or close to the ranges reported in previous studies conducted under comparable FDM printing conditions. This supports the reliability of the selected processing parameters. However, ASA exhibited tensile strength values slightly below the range commonly reported in the literature, whereas PETG surpassed typical reported values. These deviations may be attributed to factors specific to the materials, such as filament formulation, process optimisation, and interlayer bonding efficiency (Figure 8b).

Analysis of the stress–strain curves indicates that the PLA filament exhibits a higher initial slope than other materials and reaches a maximum tensile stress after a short plastic deformation region. This behaviour indicates that PLA is relatively rigid and brittle in its mechanical response. ABS specimens, on the other hand, reached maximum stress at lower elongation values and exhibited limited plastic deformation prior to failure, indicating brittle fracture behaviour under the conditions studied.

In contrast, the maximum tensile stress in PETG and PC/ABS filaments was reached after a more gradual increase, a wider plastic deformation zone formed following neck failure. The high elongation at break values presented in Table 1 (PETG: 6.27% ± 0.78; PC/ABS: 6.04% ± 0.22) quantitatively support the fact that these materials exhibit a more ductile deformation behaviour. This ductile behaviour is thought to contribute to a more homogeneous stress distribution throughout the material volume under load.

Although the slope of the elastic region in the ASA filament is relatively low, limited plastic deformation was observed following necking. Due to these properties, ASA exhibited semi-ductile mechanical behaviour among the filaments studied. This indicates that ASA is in an intermediate position in terms of both stiffness and deformation capacity.

These different deformation behaviours provide important insights into the energy dissipation capabilities of materials and the mechanisms of damage propagation. In particular, elongation prior to fracture and the width of the plastic deformation zone are critical factors in the initiation and growth of microcracks. In this context, the ductile–brittle transition trends observed in Figure 7 may be related to local stress concentrations and microstructural features such as interlayer adhesion and defect distribution. However, these interactions cannot be explained by mechanical properties alone; they must be evaluated in conjunction with printing-induced porosity, microstructural characteristics, and specimen geometry. The mechanical properties of materials, printing-induced porosity, and microstructural characteristics are influenced by the printing parameters [43,44].

### 3.2. The Breakdown Strength of 3D-Printed Materials

**Figure 9:**
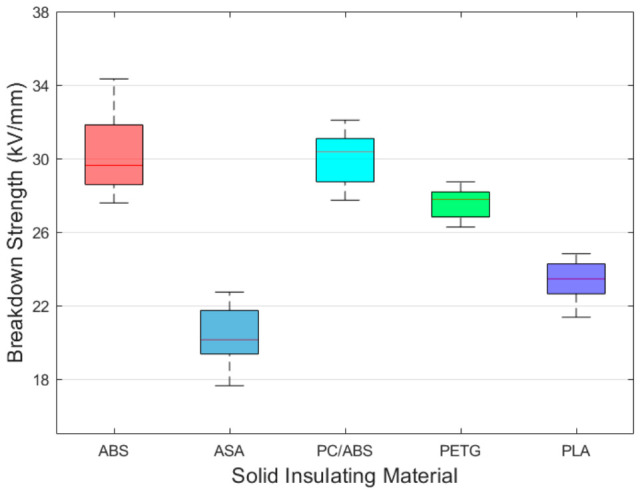
Box plot of breakdown strengths for specimens.

The test specimens were produced using temperature-controlled manufacturing processes (bed, chamber, and nozzle temperatures) for all filaments according to the manufacturers’ guidelines. Additionally, other important production factors (print speed, infill density, layer height, nozzle size) were maintained at consistent levels to maintain comparable levels of print quality. As depicted by the box plots in Figure 9, the composite materials demonstrated clear hierarchies in terms of their dielectric properties. The results from the experiments indicated that both ABS (30.28 kV/mm) and PC/ABS (30.14 kV/mm) presented the most similar high breakdown strengths, demonstrating superior insulating characteristics. Conversely, specimens made from ASA demonstrated the lowest dielectric strength of all the examined specimen types with an average of 20.35 kV/mm. The dielectric strengths of PETG (27.55 kV/mm) and PLA (23.45 kV/mm) were situated between those two extremes, as are common in the literature.

**Figure 10:**
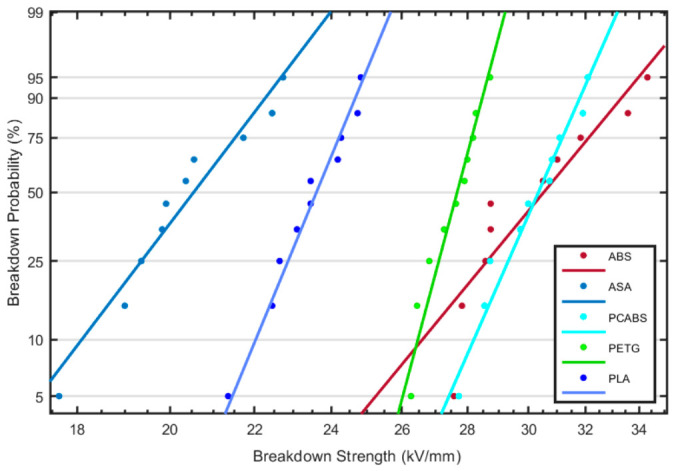
Weibull distribution of breakdown strengths for materials.

A detailed examination of the Weibull probability distribution curves presented in Figure 10 clearly reveals the critical threshold values for the risk of electrical breakdown in the materials. Statistical data indicate that the ASA material has a breakdown probability of up to 95% at a voltage level of 22 kV/mm. This shows that ASA has the weakest structure against electrical stress among the polymers tested. When a similar risk analysis is conducted, the critical voltage thresholds at which the breakdown probability reaches 95% are 24 kV/mm for PLA and 28 kV/mm for PETG. On the right-hand side of the distribution, i.e., in the higher resistance region, the PC/ABS material reaches that probability value at 32 kV/mm, whereas the ABS material exhibits strength up to 34 kV/mm, thereby indicating that it is the member of the group with the highest insulation capacity. These results show that when selecting materials for high-voltage insulation applications, not just average values but also high-probability breakdown risk limits (such as 95%) should be taken as fundamental criteria (in terms of safety margins) [15].

The data distributions shown in the graphs, together with low standard deviations, demonstrate that the test methodology exhibits high repeatability. Furthermore, given the destructive nature of the tests, the three measurements (*n* = 3) carried out on each material serve to confirm that the internal structural integrity of the polymers is maintained up to the point of breakdown. However, focusing solely on nominal puncture stress values is insufficient to fully explain the material’s engineering performance. Therefore, a thorough analysis of the observed electrical breakdown behaviour—establishing correlations with the materials’ mechanical properties and microstructural damage mechanisms—is critical to the reliability of 3D-printed insulators.

### 3.3. Comparative Evaluation of Tensile Strength and Breakdown Strength

**Figure 11:**
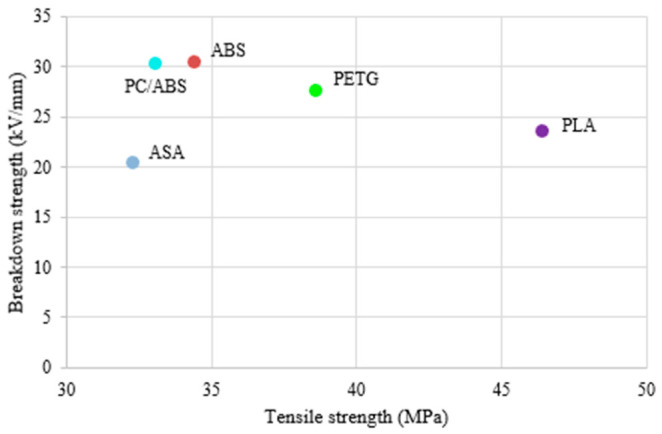
The relationship between breakdown strength and tensile strength.

Figure 11 shows the relationship between the maximum tensile stress (σ_m_) and the breakdown strength. No clear unidirectional trend is observed between these two parameters for the materials examined.

The results demonstrate that materials with high tensile strength do not necessarily exhibit higher breakdown strength in all cases. Although the ABS and PC/ABS specimens had tensile strengths than PLA and PETG, they exhibited the highest breakdown strength. In contrast, the PLA specimen, which exhibited the highest tensile strength, demonstrated an intermediate breakdown strength.

Although a linear correlation between mechanical strength and electrical insulation performance has been reported in some studies [45], the findings of this work indicate that no direct or universal relationship exists for 3D-printed insulating materials. Consequently, material selection based solely on ultimate tensile strength may be misleading for electrical insulation applications.

### 3.4. Comparative Evaluation of Elastic Properties and Breakdown Strength

**Figure 12:**
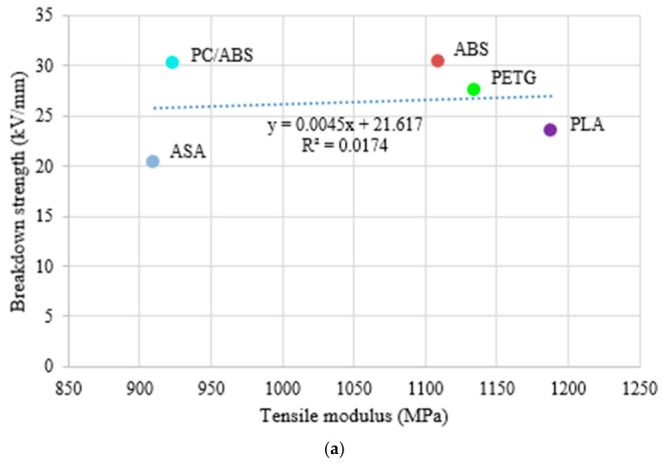

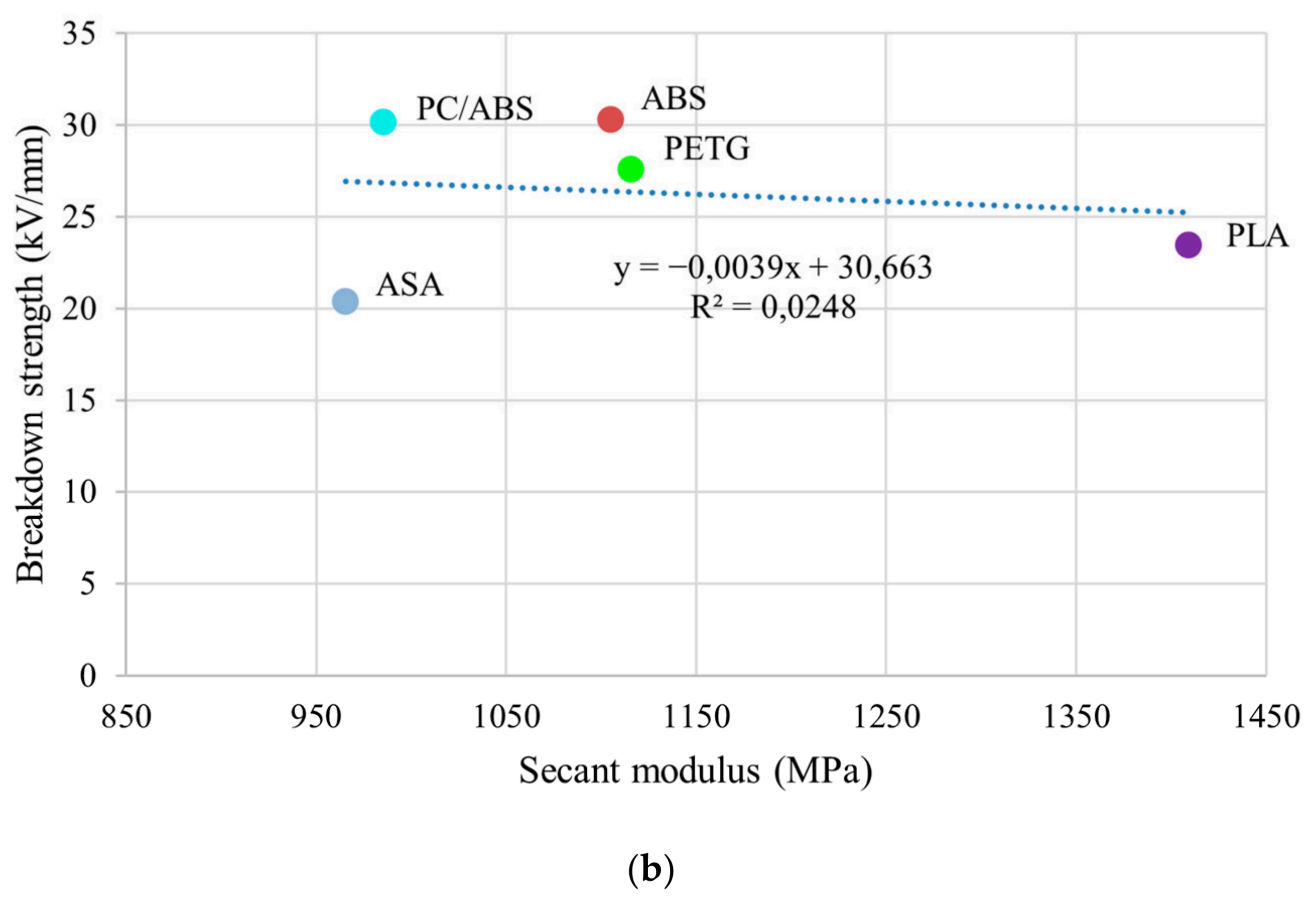
Variation in electrical breakdown strength and elastic modulus parameters: (**a**) tensile modulus; (**b**) secant modulus.

Figure 12a,b show the relationships between breakdown strength and the elastic modulus parameters (E_t_ and E_sec_). Figure 12a,b show that, within this group, PLA is the hardest and most brittle material, while ASA is the softest and least brittle. However, both materials exhibited similar breakdown strengths. Furthermore, the low R^2^ values indicate a lack of linear correlation between the elastic modulus and the breakdown strength. This suggests that, rather than elastic properties alone determining breakdown behaviour, they must be evaluated in conjunction with printing-specific microstructural factors, such as layer interfaces, internal void structures, and localised electric field concentrations [46].

### 3.5. Comparative Evaluation of Deformation Capacity and Breakdown Strength Behaviour

**Figure 13:**
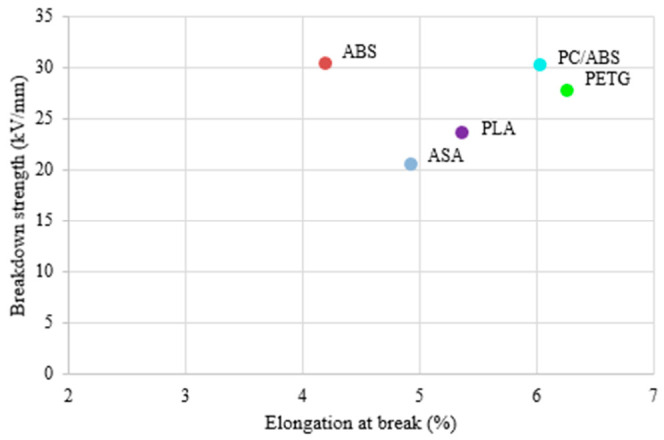
The relationship between breakdown strength and elongation at break.

Figure 13 shows the relationship between breakdown strength and elongation at break (ε^B^). Elongation at break is an important mechanical parameter reflecting a material’s capacity for plastic deformation and its resistance to damage propagation. In this case, PC/ABS and PETG exhibited high elongation values at the point of failure, making them the most ductile materials in the group, while ABS was the most brittle.

In PETG and PC/ABS specimens, relatively higher breakdown strength and elongation-at-break values were obtained. However, the ABS material exhibited the highest breakdown strength despite its low elongation at break, indicating that elongation at break alone is insufficient to explain the breakdown behaviour. This finding highlights that breakdown should be evaluated not only in terms of macroscopic deformation capacity but also in relation to microstructural factors, such as polymer chain structures, amorphous/crystalline morphology, integrity of interlayer interfaces, and resistance to localised electric field concentrations [46]. Therefore, a multiparametric approach incorporating both mechanical and microstructural factors is necessary for a comprehensive understanding of dielectric breakdown behaviour in FDM-printed polymers.

### 3.6. Quantitative Assessment of the Damage Area Using Image Analysis

**Figure 14:**
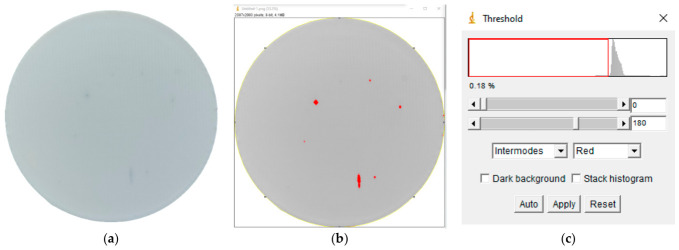
Results of image analysis of PLA specimen: (**a**) breakdown specimen, (**b**) image analysis, (**c**) result.

**Figure 15:**
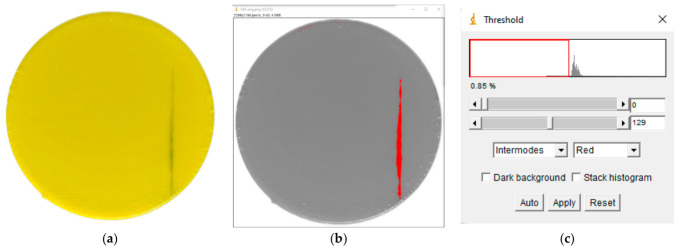
Results of image analysis of ABS specimen: (**a**) breakdown specimen, (**b**) image analysis, (**c**) result.

**Figure 16:**
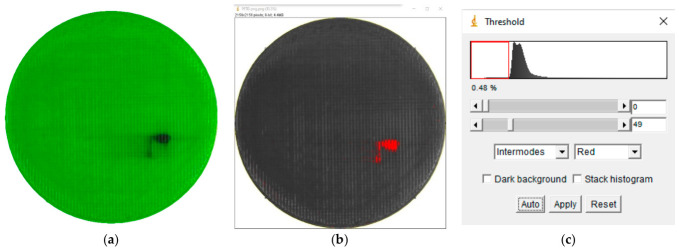
Results of image analysis of PETG specimen: (**a**) breakdown specimen, (**b**) image analysis, (**c**) result.

**Figure 17:**
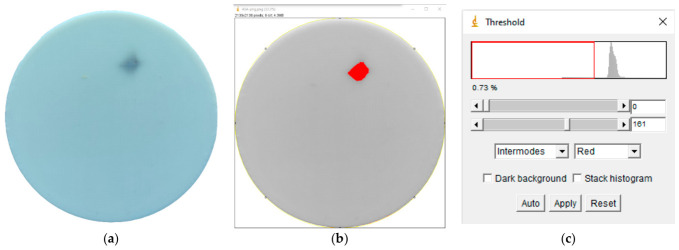
Results of image analysis of ASA specimen: (**a**) breakdown specimen, (**b**) image analysis, (**c**) result.

**Figure 18:**
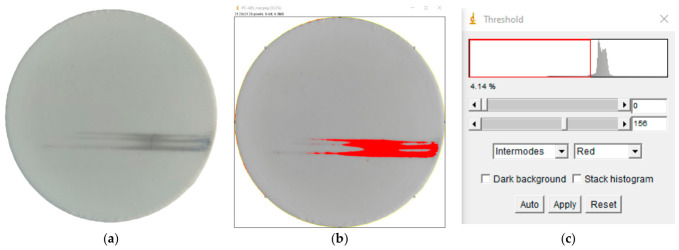
Results of image analysis of PC/ABS specimen: (**a**) breakdown specimen, (**b**) image analysis, (**c**) result.

In this section, the damage zones formed on the specimen surfaces following breakdown tests were quantitatively assessed by image analysis. The open-source ImageJ software was used for damage analysis, and all specimens were imaged at a fixed focal length of 16 cm and at a 90° angle to the surface (Figure 14a, Figure 15a, Figure 16a, Figure 17a and Figure 18a). After the images were converted to greyscale, carbonised and burnt regions were identified using the thresholding intermode method (Figure 14b, Figure 15b, Figure 16b, Figure 17b and Figure 18b), and the ratio of these areas to the total specimen surface area was calculated (Figure 14c, Figure 15c, Figure 16c, Figure 17c and Figure 18c).

Damage on the surface of the PLA specimen after the high-voltage breakdown test was examined by image analysis (Figure 14). Following the breakdown test, 0.18% of the PLA specimen’s surface area sustained damage. The data obtained indicate that the material melted locally at the point where the electrical arc formed, creating a carbonised channel. The quantitative measurements in the figure demonstrate that the damage occurring when the PLA reaches its dielectric strength limit was concentrated in a narrow area and that the material completely loses its insulating properties at this point.

Following the electrical breakdown test, approximately 0.85% of the total surface area of the ABS sample had broken down. The morphology of damage to the ABS samples shown in Figure 15 following the breakdown test is characterised by an irregular shape due to the amorphous nature of this material. Results of image processing identified that a large heat affected zone had formed around the breakdown channel as a result of thermal effects. This provides important data on the thermal stability of ABS at high voltage and the characteristics of energy distribution during electrical discharge.

Dielectric breakdown images of PETG samples illustrate how it behaves as a specimen. After the dielectric breakdown test, there was 0.48% total surface damage to the PETG sample. The amount of structural degradation and material loss at the surface of the PETG specimen, which occurs when an arc is formed, has been numerically analysed. The observed difference between images in contrast confirms the existence of micro-cracking and chemical degradation of the material radiating out from the high electrical stress region where the breakdown occurred.

The electrical breakdown test conducted on the ASA sample determines how resistant ASA is to arcing, as well as whether or not it will produce a path for surface leakage. After conducting the breakdown test, the ASA sample had approximately 0.73% of its surface area damaged. Image analysis was successively used to demonstrate the extent of the damage that occurred at the breakdown point, as well as the total surface area affected by this damage. These quantified values are essential when assessing the dependability of ASA in applications involving high voltage, as well as establishing the geometric limits of damage due to dielectric fatigue.

The breakdown damage observed in the PC/ABS alloy specimens (Figure 18) results from polycarbonate’s high heat resistance and ABS properties. Following the breakdown test, 4.14% of the surface area of the PC/ABS specimen was damaged. The results of the analysis indicate that the degree of carbonisation around the breakdown channel and the manner in which the material loses its electrical insulation differ from those observed in other polymers. Damage area data obtained from imaging techniques quantitatively confirm the limits to the electrical insulation performance of this alloy.

Image analysis studies conducted after breakdown testing have revealed that different thermoplastic polymers (PLA, ABS, PETG, ASA, and PC/ABS) exhibit distinct dielectric strengths and damage-propagation characteristics under high voltage. Comparison of quantitative data showed that PLA specimens exhibited a narrower, more localised area of damage, whereas PETG and ABS specimens showed thermal degradation from the electrical arc that spread over a wider surface area. In particular, carbonisation around the breakdown channel in the PC/ABS alloy remained within more controlled limits owing to polycarbonate’s high heat resistance, whereas ASA specimens exhibited moderate resistance to the formation of surface leakage paths. Damage area data obtained using image processing techniques confirm that the amorphous or semi-crystalline properties within the material’s internal structure are directly related to its energy absorption capacity during electrical discharge. This study numerically demonstrates that when selecting materials for applications requiring high-voltage insulation, not only the dielectric constant but also the geometric spread of structural damage following breakdown is a critical parameter.

No direct or unidirectional relationship was identified between the size of the damage area and electrical breakdown strength. However, when the damage distribution characteristics are taken into account, it was observed that in some materials breakdown was confined to small but densely carbonised regions, whereas in others it resulted in superficial damage that spread over wider areas. This finding shows that breakdown must be evaluated not only in terms of its maximum resistance value but also in relation to the material’s electro–thermo–mechanical response and to the mechanism of damage propagation. When the results of the image analysis were considered alongside the mechanical and electrical data presented in the previous sections, it is evident that breakdown in 3D-printed polymers is a multi-factor process and that material selection based on a single performance criterion is insufficient.

## 4. Conclusions

In this study, the mechanical properties, dielectric breakdown strength, and surface damage characteristics of ABS, PLA, PETG, ASA, and PC/ABS thermoplastic polymers produced via FDM-based 3D printing were comparatively evaluated for potential electrical insulation applications. Breakdown tests were conducted according to IEC 60243 standarts, while tensile tests were performed according to ASTM D638-14 standards. Post-breakdown surface damage was quantitatively analysed using ImageJ image processing software. The main findings are summarised below:

- PLA specimens exhibited the highest tensile strength (46.44 MPa) and the lowest surface damage ratio (0.18%), although their dielectric breakdown strength was comparatively lower.
- ABS specimens exhibited the highest dielectric breakdown strength (30.28 kV/mm), despite comparatively low tensile strength (34.46 MPa) and restricted elongation at break.
- ASA specimens showed the lowest dielectric breakdown strength (20.35 kV/mm) and generally lower mechanical performance.
- PC/ABS specimens exhibited the highest surface damage ratio (4.14%). This indicates greater structural degradation under high-voltage conditions.
- PETG specimens provided the most balanced overall performance, combining relatively high tensile strength (38.63 MPa), the highest elongation at break (6.27%), and strong dielectric breakdown resistance (27.55 kV/mm). These characteristics identify PETG as a promising candidate among the investigated materials for 3D-printed electrical insulation applications.
- No direct or unidirectional relationship was identified between tensile strength and dielectric breakdown strength. This finding suggests that material selection for 3D-printed electrical insulation components should not rely solely on mechanical strength, but rather on a multi-parameter assessment that also considers electrical performance, deformation capacity, and damage behaviour.

Overall, the results suggest that the dielectric breakdown behaviour of 3D-printed polymers may be influenced by multifactorial interactions involving material composition, deformation behaviour, structural integrity and printing-induced defects. Therefore, careful consideration of printer settings, material selection and process parameters is essential for the successful production of FDM-based electrical insulation components.

This study contributes to a broader comparative understanding of the electrical insulation performance of commonly used FDM thermoplastics. Furthermore, it establishes a valuable basis for future investigations into how printing parameters influence dielectric behaviour and overall insulation performance.

## Figures and Tables

**Table 1:** Technical specifications of the filaments [31].

Specifications		PLA	ABS	PETG	ASA	PC/ABS
Density (g/cm^3^)	ISO 1183	1.23	1.03	1.28	1.06	1.13
Melt Flow Index (g/10 min)		17.3	5.2	20	22	12
Heat Deflection Temperature (°C)		55	95	80	95	120
Glass Transition Temperature (°C)		55–60	95–105	80–85	100–105	125–135
Surface Resistance (Ohm/sq)		>10^12^	>10^12^	>10^12^	>10^12^	>10^12^

**Table 2:** Printing parameters used in a 3D printer according to filament type.

Printing Parameter	PLA	ABS	PETG	ASA	PC/ABS
Nozzle diameter (mm)	0.4	0.4	0.4	0.4	0.4
Layer height (mm)	0.2	0.2	0.2	0.2	0.2
Infill density (%)	100	100	100	100	100
Infill pattern	Rectilinear	Rectilinear	Rectilinear	Rectilinear	Rectilinear
Nozzle temperature (°C)	220	283	240	275	283
Plate temperature (°C)	60	110	80	100	110
Printing chamber temperature (°C)	-	53	-	53	53
Internal layer printing speed (mm/s)	50	50	50	50	50
Internal layer infill printing speed (mm/s)	105	105	105	105	105
External wall printing speed (mm/s)	200	200	200	200	200
Internal wall printing speed (mm/s)	300	300	300	300	300
Printing cooling fan speed (%)	100	-	100	100	-
Build plate adhesion type	Brim	Brim	Brim	Brim	Brim

**Table 3:** Drying temperatures and times for PLA, ABS, PETG, ASA and PC/ABS filaments.

	PLA	ABS	PETG	ASA	PC/ABS
Temperature (°C)	50	65	60	65	65
Drying time (h)	6	10	6	10	10

**Table 4:** Mechanical properties of specimens produced by 3D printing (*n* = 5).

	PLA	ABS	PETG	ASA	PC/ABS
E_t_—Tensile Modulus (MPa)	1188.41 ± 22.17	1109.30 ± 80.32	1134.31 ± 55.44	910.48 ± 112.06	924.05 ± 74.75
E_Sec_—Secant Modulus (MPa)	1409.18 ± 123.06	1105.40 ± 50.82	1115.72 ± 27.92	965.63 ± 44.93	985.26 ± 65.77
s_M_—Tensile Strength (MPa)	46.44 ± 0.36	34.46 ± 0.42	38.63 ± 0.76	32.32 ± 0.60	33.10 ± 0.77
ε_M_—Elongation at Tensile Strength (%)	3.24 ± 0.10	3.11 ± 0.07	3.80 ± 0.04	3.70 ± 0.09	4.29 ± 0.04
s_B_—Stress at Break (MPa)	39.65 ± 1.53	32.10 ± 0.89	28.55 ± 3.85	28.76 ± 1.29	25.04 ± 0.72
ε_B_—Elongation at Break (%)	5.37 ± 0.15	4.21 ± 0.45	6.27 ± 0.78	4.94 ± 0.36	6.04 ± 0.22

## Data Availability

The original contributions presented in this study are included in the article. Further inquiries can be directed to the corresponding author.
